# Myocardial fibrosis with T1 mapping and right ventricular performance in pulmonary hypertension

**DOI:** 10.1186/1532-429X-15-S1-E102

**Published:** 2013-01-30

**Authors:** Ines Garcia-Lunar, Pablo Pazos, Eduardo Pozo, Claudia Calcagno, Sarayu Ramachandran, Charles M Adapoe, Ajith Nair, Adam Jacobi, Valentin Fuster, Javier Sanz

**Affiliations:** 1 Mount Sinai School of Medicine, New York, NY, USA

## Background

Pulmonary hypertension (PH) leads to progressive right ventricular (RV) dilatation, hypertrophy, and systolic dysfunction. PH is also associated with the presence of late gadolinium enhancement in the interventricular septum (IVS). Post-contrast T1 mapping is a previously validated non-invasive technique for the quantification of extracellular volume increase as a surrogate of diffuse interstitial fibrosis. The aim of our study was to evaluate the presence of septal fibrosis in PH patients with T1 mapping, and to investigate potential correlations with RV performance and hemodynamic status.

## Methods

24 patients with known or suspected PH of various etiologies (excluding left heart disease) were included in the study. Cardiac magnetic resonance (CMR) was performed on a 3-T scanner and the IVS T1 time was quantified offline by an independent and blinded observer on a Look-Locker sequence acquired 5-15 minutes after the infusion of 0.19±0.03 mmol/kg of gadopentetate dimeglumine. End-diastolic RV free wall thickness was measured on a cine sequence at the left ventricular outflow tract view. Twenty (83%) patients additionally underwent right heart catheterization. Using Pearson r coefficients, septal T1 times were correlated with RV volumes and ejection fraction as derived from CMR, and cardiac output and pulmonary pressures and vascular resistance obtained invasively.

## Results

**Figure 1:**
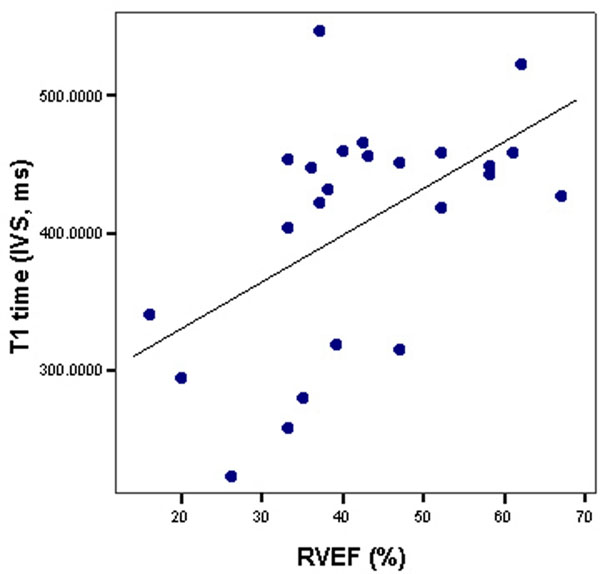
Correlation between T1 time and right ventricular ejection fraction.

The mean age was 53.8±11.9 years and 29.2% of the patients were males. PH (mean pulmonary artery pressure >25 mmHg) was present in 92%. In patients with PH, the etiology was pulmonary arterial hypertension in 63.6% (36.4% idiopathic PH), connective tissue disease in 9.1%, lung disease in 18.2%, and miscellaneous in 9.1%. Patients with shorter IVS T1 times showed more severe RV dilatation, RV hypertrophy and systolic dysfunction (Figure 1), higher invasive pulmonary vascular resistances, and lower cardiac output (Table 1). Most of these relationships remained statistically significant after adjusting the myocardial T1 time by skeletal muscle T1 time (not shown).

**Table 1:** Correlation of IVS T1 time with CMR and right heart catheterization measurements.

	RVEF	RVEDVi	RVESVi	RV wall thickness	mPAP	PVR	CO
r (Pearson)	0.53	-0.32	-0.44	-0.44	-0.109	-0.45	0.44

P	0.007	NS	0.029	0.032	NS	0.046	0.049

CO: cardiac output; RVEDVi: right ventricular end-diastolic volume index; RVEF: right ventricular ejection fraction; RVESV: right ventricular end-systolic volume index; mPAP: mean pulmonary artery pressure, PVR: pulmonary vascular resistance.

## Conclusions

A reduction in post-contrast T1 times in the IVS, possibly indicating increased interstitial fibrosis, correlates with RV structural and functional impairment and hemodynamic severity in PH. T1-mapping may constitute a novel approach for the evaluation of cardiac adaptation to pressure overload.

## Funding

None.

